# Thyroid hormones – obesity and metabolic syndrome

**DOI:** 10.1186/1756-6614-6-S2-A5

**Published:** 2013-04-05

**Authors:** Elżbieta Bandurska-Stankiewicz

**Affiliations:** 1 Clinic of Endocrinology, Diabetes and Internal Diseases, Department of Internal Diseases, University of Warmia and Mazury in Olsztyn, Poland; 2 Department of Medical Science, University of Warmia and Mazury in Olsztyn, Poland

## 

Recently there has been increased interest in the association between thyroid function and obesity. Based on the notion that triiodothyronine (T3) controls metabolic and energy homeostasis and influences body weight, thermogenesis, lipolysis and metabolism of cholesterol, and that thyroid-stimulating hormone (TSH) via receptors in fat tissue, induces differentiation of preadipocytes in to adipocites and expansion of adipose tissue (adipogenesis), thyroid function has been extensively investigated in obese adults. An elevated level of TSH with normal peripheral thyroid hormone concentration suggesting sub-clinical hypothyroidism has been consistently found in obese subjects. Several mechanisms leading to hyperthyrotropinemia have hypothesis including sub clinical hypothyroidism caused by iodine deficiency, autoimmune thyroiditis or mutations in TSH-R gene. TSH production is also regulated by neurotransmitters and hormones that influence body weight such as neuropeptide Y and alfa-melanocyte-stimulating hormone related peptide, that innervate hypophysiotropic TRH neurons. These neurotransmitters and hormones are also influenced by leptin. Thyrotropin also directly induces the synthesis and release of adipokines. Some of them control appetite by acting on the brain. Metabolic syndrome (MS) is clustering obesity, hypertension, dyslipidemia and insulin resistance. MS is a status where most features of hypothyroidism can be seen. TSH increase was shown to be associated with increased cholesterol and triglycerides and with decreased HDL-C. Thyroid hormones are important determinants of glucose homeostasis. Increased thyroid hormone levels impair the ability of insulin to suppress hepatic glucose production and increase glucose uptake in muscles. An association between TSH and fasting insulin and insulin sensitivity has been reported in adults with obesity. The increased TSH and peripheral hormone levels, which are usually in the upper normal range in obese subjects may be adaptation process to increase energy expenditure in order to reduce further weight gain. The changes of thyroid hormones concentration may be regarded as a consequence rather than a cause of obesity. MS is cluster of metabolic abnormalities with insulin resistance as a major component. The patients with MS have significantly increased thyroid volume and nodule prevalence and insulin resistance is an independent risk factor for nodule formation. The prevalence of insulin resistance would be an important risk factor for developing thyroid cancer and some other non-thyroid cancers.

